# Turner Syndrome With Central Precocious Puberty During Growth Hormone Therapy: Combined Treatment With a Gonadotropin-Releasing Hormone Analog

**DOI:** 10.7759/cureus.80107

**Published:** 2025-03-05

**Authors:** Katsunori Tanaka, Midori Tagaya

**Affiliations:** 1 Department of Pediatrics, National Hospital Organization Higashi-Ohmi General Medical Center, Higashiomi, JPN

**Keywords:** central precocious puberty (cpp), gonadotropin-releasing hormone (gnrh) analog, growth hormone therapy, short stature, turner syndrome (mosaic)

## Abstract

Turner syndrome (TS) is a chromosomal disorder characterized by short stature and gonadal dysgenesis. However, precocious puberty is rarely observed in TS, and its optimal management remains unclear. We report a case of a 10-year-old girl with TS and a 45,X/46,XX mosaic karyotype who presented with menarche. She had been receiving growth hormone (GH) therapy since the age of four years and six months. Laboratory and imaging findings confirmed central precocious puberty (CPP), and gonadotropin-releasing hormone analog (GnRHa) therapy was introduced alongside GH therapy. Treatment with GnRHa continued until 11 years and 11 months, while GH therapy was maintained until 14 years and 11 months. The patient ultimately achieved a final height of 140.0 cm (-3.3 SD), within the target height range. This case highlights the rare occurrence of CPP in TS and demonstrates the potential impact of combined GH and GnRHa therapy on growth outcomes. Further studies are needed to establish the optimal treatment strategies, including the appropriate timing and duration of therapy for TS patients with CPP.

## Introduction

Turner syndrome (TS) is a chromosomal disorder characterized by partial or complete monosomy of the X chromosome, with an estimated incidence of one in 2,000 live births. It is associated with various congenital anomalies, including short stature and gonadal dysgenesis, often necessitating hormone-based interventions. Growth hormone (GH) therapy is widely used to improve the final adult height in patients with TS. While primary ovarian insufficiency is a hallmark of TS, approximately 30% of affected individuals experience spontaneous puberty despite ovarian dysfunction [1,2].

In rare cases, TS may be complicated by precocious puberty, defined as the early activation of sex hormone production, leading to the premature development of secondary sexual characteristics. The prevalence of precocious puberty varies from 29 to 92 per 100,000 individuals, with a higher frequency in females. Precocious sexual maturation is classified into two types: central precocious puberty (CPP), caused by premature activation of the hypothalamic-pituitary-gonadal axis, and peripheral precocious puberty, which results from hormone secretion independent of gonadotropin stimulation. Treatment primarily involves gonadotropin-releasing hormone analog (GnRHa) therapy, which suppresses premature activation of the hypothalamic-pituitary-gonadal axis, thereby delaying puberty and allowing for a longer growth period [3-5]. However, treatment may not be necessary if psychosocial concerns are minimal, and the predicted final height remains within an acceptable range [3]. The therapeutic effect on final height is generally limited in girls who develop breast enlargement after the age of six or in cases of slowly progressing precocious puberty [6].

Reports of TS complicated by precocious puberty are rare, and optimal management strategies remain unclear [7-9]. The coexistence of precocious puberty in TS can accelerate bone maturation, potentially compromising final adult height. Therefore, a combined approach using GH therapy and GnRHa treatment may be considered to optimize growth potential and prevent premature epiphyseal closure.

Here, we report a case of a girl with TS who developed CPP and was treated with a combination of GH therapy and GnRHa therapy. We discuss the clinical course and management strategies for this rare condition.

## Case presentation

A 10-year-old girl presented with early menarche as her chief complaint, raising concerns about precocious puberty. She had been diagnosed with TS at the age of three after being identified with short stature at a routine health check-up, and subsequent genetic analysis confirmed a 45,X/46,XX mosaic karyotype.

She was born to unrelated parents at 38 weeks and 5 days of gestation, with a birth weight of 2,290 g (-1.92 SD) and a birth length of 47.0 cm (-0.82 SD). There were no congenital cardiac, auditory, or renal abnormalities, and thyroid function was within the normal range. GH therapy (Somatropin: 0.35 mg/kg/week) was initiated at four years and six months of age due to short stature. At treatment initiation, her height was 90.0 cm (-3.2 SD) and weight was 11.0 kg (-2.4 SD). Her parental heights were 155.0 cm (father) and 143.3 cm (mother), yielding a target height of 142.65 ± 8 cm.

At 10 years of age, laboratory findings revealed elevated levels of luteinizing hormone (LH) (2.4 mIU/mL), follicle-stimulating hormone (FSH) (3.8 mIU/mL), and estradiol (36.5 pg/mL). A gonadotropin-releasing hormone (GnRH) stimulation test showed a peak LH of 57.4 mIU/L and peak FSH of 20.7 mIU/L, confirming central activation of the hypothalamic-pituitary-gonadal axis. Brain and abdominal MRI ruled out adrenal or intracranial tumors, revealing normal ovaries and a uterus of near-adult size. Bone age, assessed using the radius-ulna-short (RUS) bone method, was 13.2 years, which was significantly advanced compared to her chronological age. At this time, her height was 131.6 cm (-0.8 SD) and weight was 34.9 kg (+0.3 SD).

Based on these findings, a diagnosis of CPP was made. The key diagnostic criteria included the occurrence of menarche before 10 years and 6 months of age, a bone age that was more than two years and six months ahead of her chronological age, and elevated baseline and stimulated levels of LH, FSH, and estradiol. Additionally, imaging studies did not detect any brain or adrenal tumors, and there was no history of medication use or excessive consumption of foods containing high levels of sex steroids.

**Figure 1:**
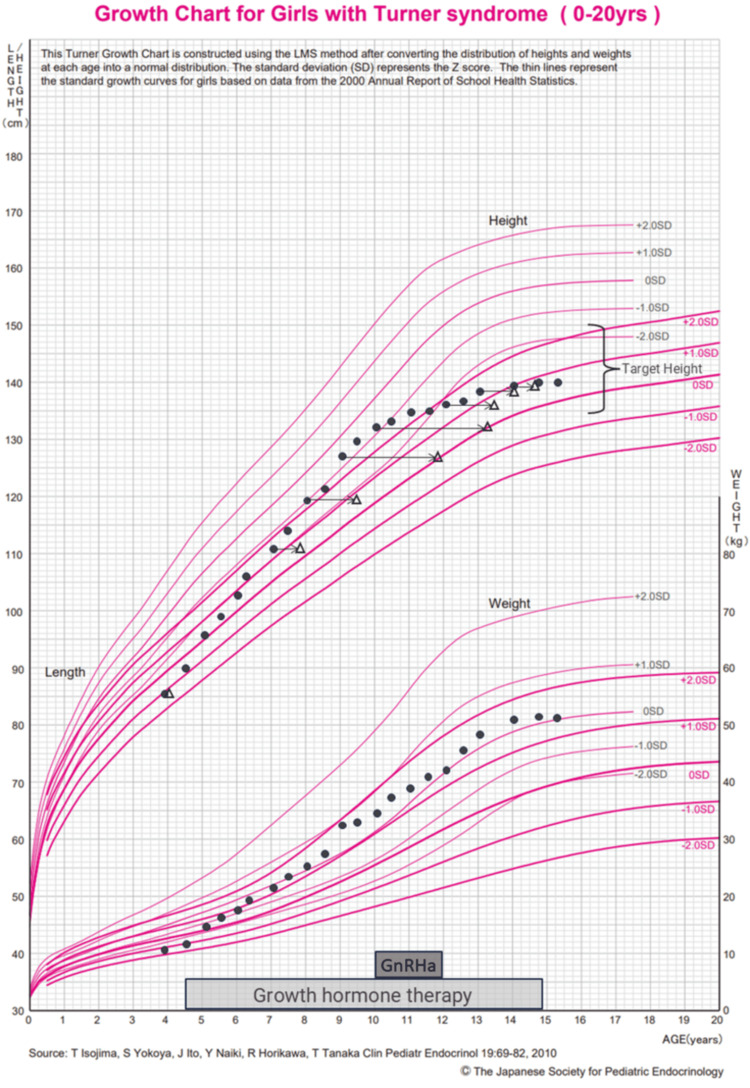
Growth chart This growth chart was created using reference data from the Japanese Society for Pediatric Endocrinology [10]. The black circles represent actual measurements of height and weight, while the white triangles indicate bone age. The arrows illustrate the degree of bone maturation advancement. The lower part of the figure shows the treatment periods for gonadotropin-releasing hormone analog (GnRHa) therapy and growth hormone (GH) therapy. A notable increase in growth velocity was observed following the initiation of GH therapy at four years and six months of age. However, after the introduction of GnRHa therapy at 10 years of age, the acceleration of bone maturation was suppressed, which contributed to prolonged growth potential. These trends are reflected in the figure, illustrating the impact of each therapy on the patient’s growth trajectory. Permission for the use of this figure has been obtained from the Japanese Society for Pediatric Endocrinology.

To optimize growth potential and delay bone maturation, GnRHa therapy was initiated at 10 years of age, in addition to continued GH therapy. GnRHa therapy was discontinued at 11 years and 11 months, while GH therapy was maintained until 14 years and 11 months. The clinical course of height, weight, bone age, and treatment duration are summarized in Figure 1 [10].

At the end of treatment, her final height was 140.0 cm (-3.3 SD), which fell within the expected range based on parental height. Menstruation resumed at 12 years and 3 months. At 14 years and 7 months, bone mineral density (BMD) assessment using the DIP method showed a ΣGS/D of 2.77 mmAL, corresponding to 97% of the mean for young adult females, suggesting no adverse impact on bone health. TS patients are known to have an increased risk of metabolic syndrome. However, throughout the course of follow-up, she did not exhibit hypertension, impaired glucose tolerance, or dyslipidemia. Continued follow-up is planned to monitor for potential metabolic complications in the future.

## Discussion

This case presented a patient with TS complicated by CPP. In addition to GH therapy for short stature, we introduced GnRHa therapy to manage CPP. GH therapy effectively promoted height growth, while GnRHa therapy appeared to suppress the acceleration of bone maturation. However, since treatment was initiated at 10 years of age, the improvement in final adult height may have been limited. Nevertheless, the patient ultimately achieved a height within the target range.

The occurrence of CPP in TS is extremely rare. While several case reports have documented this condition, all reported cases of TS with CPP have exhibited a mosaic karyotype [7-9,11-14]. Similarly, our patient also had a mosaic karyotype.

A few reports have described cases of TS with CPP treated with a combination of GH therapy and GnRHa therapy [7-9,11]. However, only one report provided details on final adult height [9]. In that case, GH was administered considering its effects on anti-Müllerian hormone levels and ovarian function rather than for short stature. In contrast, our case involved a patient who had already been receiving GH therapy for short stature and later developed CPP, necessitating the addition of GnRHa therapy. We documented the treatment course up to final height and demonstrated the effects on height promotion and the suppression of bone maturation acceleration.

The patient’s final height was 140.0 cm (-3.3 SD). When compared with previously reported outcomes, TS patients receiving GH therapy generally achieve an average final height of approximately 143-146 cm, while those without GH therapy often have an untreated final height of around 140 cm [15-17]. Given that her final height was lower than the average for GH-treated patients, it is possible that the presence of CPP and the delayed initiation of GnRHa therapy contributed to the reduced height gain.

Long-term GnRHa therapy may delay the onset and progression of secondary sexual characteristics and raise concerns regarding reduced bone mineral acquisition. Since TS is often accompanied by gonadal insufficiency, careful monitoring is required. In our case, we confirmed the progression of secondary sexual characteristics following treatment and found no decrease in BMD. Although some studies have reported the effectiveness of GH and GnRHa combination therapy [18], further accumulation of cases is needed to clarify the most appropriate treatment regimen, including the optimal timing and duration of therapy in TS.

## Conclusions

This case involved a patient with TS complicated by CPP. In addition to GH therapy for short stature, GnRHa therapy was introduced to manage precocious puberty. The patient ultimately achieved a final height of 140.0 cm (-3.3 SD), which was within the target height range of 134.65-150.65 cm. The combination of GH and GnRHa therapy appeared to help optimize growth potential by suppressing the acceleration of bone maturation. Further studies are needed to establish the most appropriate treatment strategies, including the timing and duration of therapy, for TS patients with precocious puberty.
